# Young people on social media in a globalized world: self-optimization in highly competitive and achievement-oriented forms of life

**DOI:** 10.3389/fpsyg.2024.1340605

**Published:** 2024-07-05

**Authors:** Stephen Butler

**Affiliations:** Department of Psychology, University of Prince Edward Island, Charlottetown, PE, Canada

**Keywords:** social media, extrinsic values, market-driven identity, self-optimization, globalization, capitalism

## Abstract

Research investigating young people’s social media use has been criticized for its limited theoretical foundations and scope. This paper elaborates young people’s social media activity from a socio-ecological evolutionary perspective (SEE), where young people’s online exchanges cannot be divorced from the highly competitive and achievement-oriented modern market cultures in which they live. In highly competitive and achievement-oriented forms of life, young people’s social media environments are often constituted as dynamic and evolving extrinsically oriented ecological niches that afford for status and identity enhancement while also affording for peer approval, belongingness, and self-worth nested within, and subordinate to, these higher-order affordances. The extrinsic value organization of social media platforms that serve young people’s status and identity-enhancement are embodied by a community of mutually interdependent criteria that are evolutionary-based, developmentally salient, and market-driven: physical attractiveness, high (educational and extracurricular) achievements, and material success. Young people’s online signaling of these interdependent extrinsic criteria affords for status-allocation and self-enhancement, where each criteria becomes an arena for social competition and identity formation, enabling young people to build personal and optimal models of social success congruent with their own interests and abilities. Young people’s status and identity enhancing signaling of these extrinsic criteria is moving toward increasingly idealized or perfect embodiments, informed by accelerating, short-term positive feedback processes that benefit from the technological affordances and densely rewarding peer environments instantiated on social media.

## Introduction

The phenomenal growth of social media over the last two decades is embedded in two key developments of the 21st century: globalization (Jensen and Arnett, 2012; McKenzie, 2019) and the spread of modern, market-based economies across the world (Greenfield, 2009; Milner and Mukherjee, 2009; Milanovic, 2019). These twin developments have dramatically increased the degree and intensity of connections among different cultures and world regions and propelled the spread of an individualistic and extrinsically oriented market-based value structure throughout much of the world (Greenfield, 2013; Santos et al., 2017; Velichkovsky et al., 2019) Consequently, young people’s prolific social media use is contextualized and shaped by the extrinsic value structure and the highly competitive and achievement oriented modern market cultures in which they live (Butler, 2018, 2021).

This paper examines young people’s social media use from a socio-ecological evolutionary approach (SEE approach). In doing so, it addresses repeated calls for interdisciplinary theories and conceptual models to guide social media research with young people that are context-sensitive, and that consider how individual, interpersonal, technological, and broader socio-cultural factors interact to influence young people’s social media activities and their developing identities and well-being (Nesi et al., 2020; Orben, 2020; Sarmentio et al., 2020; Tibber and Silver, 2022). Following a brief introduction to the SEE framework, delimiting its applicability to social media research with young people, the paper is divided into two main sections. The first section draws on research from the evolutionary and developmental sciences to detail how young people’s social media activity has quickly become part of the socio-ecology of competition and status-seeking characteristic of complex modern market cultures. In the highly competitive and achievement-oriented forms of life characteristic of modern market cultures, young people on social media may optimize their status and identity enhancing signaling of their culturally valued competencies and characteristics to attain stage-salient status, and to build alliances with their peers, thus beginning to secure access to valuable social, psychological, and material resources. The second section draws on niche construction and cultural affordance research to describe how young people’s online status and identity-enhancing signaling allow them to develop personal and optimal narratives of social status and success, while also attending to intrinsic needs for recognition, belongingness, and self-worth. The paper concludes with a summary of the SEE model applied to young people’s social media use, identifying its limitations while proposing how this framework may contribute to future research into young people’s online activities.

## Young people online: a socio-ecological evolutionary framework

The SEE framework approaches young people’s prolific engagement with social media, particularly as their activities pertain to identity formation and self-making during adolescence and emerging adulthood, by considering how socio-cultural factors interact with their evolved psychology (Davis and Arnocky, 2020). This conceptual framework suggests that psychological processes arise from evolutionarily shaped biological potentials that become attuned to the particular cultural meaning systems that characterize modern market cultures (Heine, 2010). The SEE model proposes that the interaction of young people’s evolutionary based motives for status-seeking, and the continuing development of individualistically oriented market-based societies that accentuate extrinsic values, has led young people and their broader social ecologies to invest in highly competitive and achievement-oriented forms of life. Young people’s status and identity enhancing signaling in their extrinsically oriented social media environments is co-extensive with their ongoing participation in highly competitive and achievement-oriented forms of life, which are part of the intensifying competition (Naidoo, 2016; Musselin, 2018) and increasing prominence of extrinsic values in modern market cultures (Schwartz, 2007; Greenfield, 2009; Twenge and Kasser, 2013).

The SEE model is applicable to young people’s active use of social media to develop and enhance their reputations and status, while concurrently developing social connections, two of the most central functions associated with social media use (Liu et al., 2016; Bekalu et al., 2019). Extrinsically oriented social media ecologies often involve peer audiences that are highly responsive to young peoples’ status and identity-enhancing signaling of extrinsic value metrics or market-driven criterion (MDC; Butler, 2018, 2021), namely images of physical attractiveness, high academic and extra-curricular achievements, and material success (e.g., status-enhancing material possessions). Young people who “passively” scroll through the profiles of their peers that implicate their developing identities, sometimes leading to upward and judgmental social comparisons around MDC such as their body image and material possessions, would also be relevant (e.g., Vogel et al., 2015; Verduyn et al., 2020. Yang et al., 2021). The SEE framework is not applicable to young people’s activities on social media such as building social connections with small groups of real-world intimate friends (Pouwels et al., 2021) or creating specific accounts on social media platforms to engage in forms of self-expression that are not so governed by audience members (e.g., a Finsta-fake Instagram account, Kang and Wei, 2020). Certainly, the SEE conceptualization cannot be generalized to young people’s social media research on the whole.

**Figure 1:**
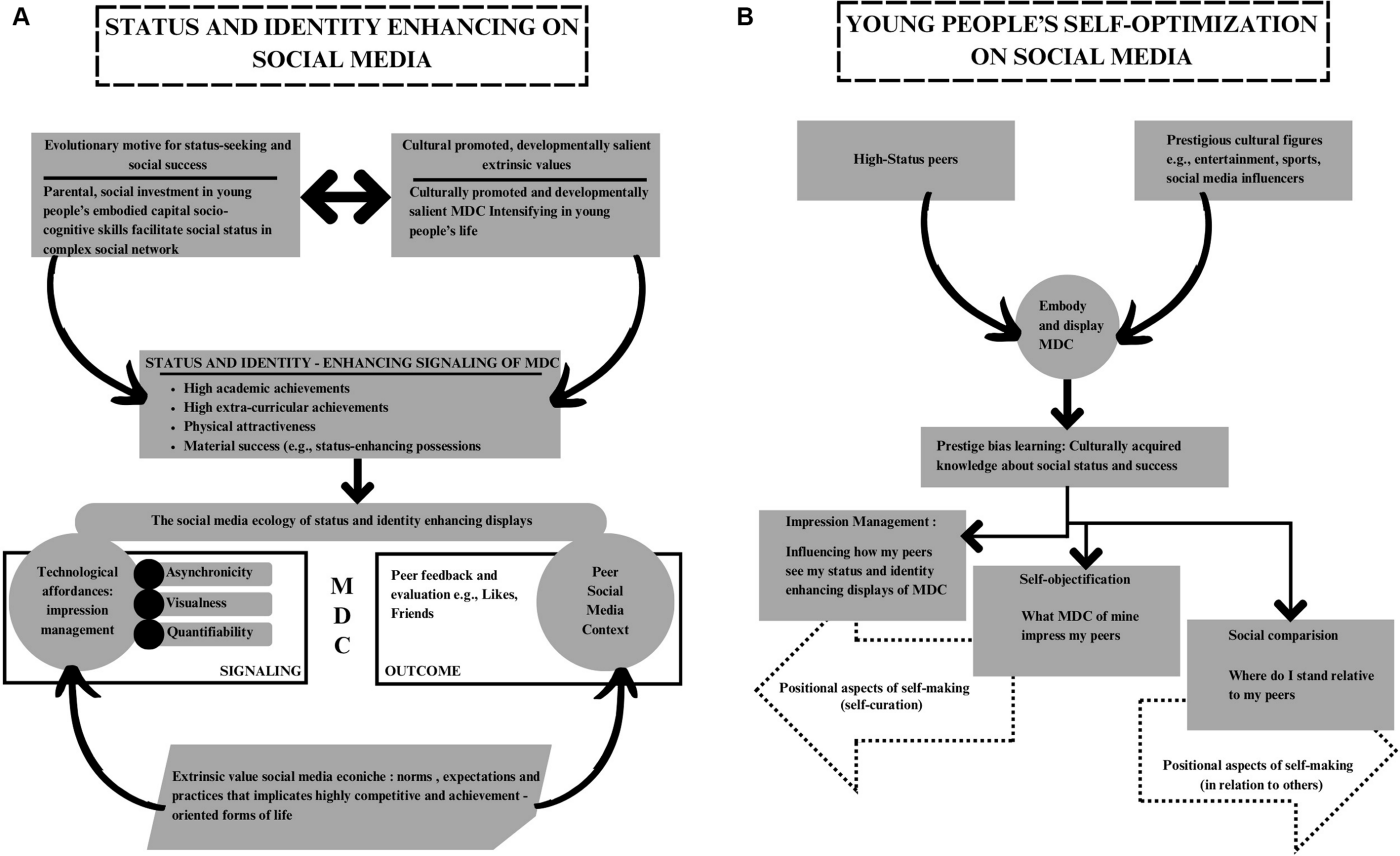
**(A)** Young people’s status and identify-enhancing signaling on social media. **(B)** Young people’s self-optimization on social media: applying their cultural learning and socio-cognitive capacities.

Notwithstanding these foci and constraints, the SEE framework addresses the dynamic interface between young people’s social media use and their identity and self-making with a conceptualization that explicitly details relationships between young people’s self-processes (e.g., impression management, self-objectification, social comparison), their use of social media’s technological architecture, and the interpersonal and socio-cultural contexts in which their online activities are embedded. The highly appealing and complex young person-social media ecology coupling is also considered; namely that young people’s mediated online communications afford for multiple motivations and needs, often concurrently, and is governed by highly responsive peer audiences that provide stage-salient feedback and evaluation to a degree previously unavailable in modern communicative environments. The interactions between young people’ motivations and psychological needs, their adaptation of the technological affordances of the medium in the service of identity and self-making, and the capacities of the medium to provide peer evaluation, feedback and reinforcement, result in potentially transformative influences on young people’s development (Nesi et al., 2018a, 2018b) and well-being (Tibber and Silver, 2022). Figure 1A maps the SEE framework and young people’s status and identity-enhancing signaling on social media that is elaborated in this paper.

### Part A: Status and identity-enhancement in highly competitive and achievement-oriented forms of life

#### Definitions

As noted above, the SEE framework proposes that the interaction of evolutionary based motives for status-seeking, and the continuing development of individualistically oriented market-based societies that accentuate extrinsic values, has led to sustained investment in highly competitive and achievement-oriented forms of life. The notion of a *form of life* is adopted from the literature on affordances and embodied cognition (Rietveld and Kiverstein, 2014), which is defined as “a set of behavioral patterns, relatively robust on socio-cultural and biographical time scales, which is characteristic of a group or population” (Ramstead et al., 2016, p. 4). Human habits, customs, beliefs, attitudes, feelings, desires, and ways of doing things that prevail in everyday life encompass a form of life (Van Dooren, 2014). Different human communities, cultures and societies develop different ways of investing in and relating to the socio-material environment, constituting different forms of life (Ramstead et al., 2016).

Young people’s participation in highly competitive and achievement-oriented forms of life engenders a distinct configuration of identity- and self-making, technology use, and engagement with peer and broader socio-cultural environments. Unending status competitions in the pursuit of social status and social success are integral to highly competitive and achievement-oriented forms of life (Turke, 1990; Alexander, 2008; Summers and Crespi, 2013; Winegard et al., 2018). In these socio-ecologies, young people become invested in their self-optimization and in the positional aspects of self-making, fostering their ability to establish their social standing and develop socially and economically productive lives in complex, modern market cultures.

*Self-optimization* may be defined as the acquisition and development of the skills, means, and resources necessary to enhance the probability of attaining social status and success (Baltes, 1997). Self-optimization is developmentally contingent and is particularly operative from mid-adolescence through the transition to adulthood (Carstensen et al., 2003; Napolitano et al., 2011). Across this developmental arc, young people’s attempts to optimize their desired life outcomes involves their heightened sensitivity to extrinsic values and goals that overshadows the attention and cognitive resources that they may deploy toward intrinsic goal-pursuit centered on relationships and emotional well-being (Baltes, 1997; Carstensen et al., 1999, 2003; Crone and Dahl, 2012). Young people become increasingly future-oriented, allocating considerable resources toward obtaining the knowledge and skills that will allow them to meet modern market environmental demands and maximize their life chances to succeed (Carstensen et al., 2003).

Self-optimizing strategies that facilitate attainment of desired life outcomes include modeling successful others, acquiring new knowledge and skills, investing more time and effort into goal pursuit, and persistently practicing new skills (Baltes, 1997). Developmentally, young people’s *self-optimization* straddles their progression from society’s investment in their maturation, through partnering, parenting, education and healthcare, referred to as outside-in investments, toward young people’s investments in their own economic productivity and life satisfaction, referred to as inside-out investments, with benefits to society as a whole (Worthman, 2012). Notwithstanding these broad developmental and cultural trends, young people’s self-optimizing occurs in differentiated and complicated social environments where opportunities and access to resources are unequally distributed (Wilkinson and Pickett, 2009, 2019) and are often stratified based on region, race, ethnicity, gender, and socio-economic differences (Worthman and Trang, 2018). These foundational social and economic inequalities, which are believed to be an inescapable part of modern market economies (Melamed, 2015), may lead to uneven life chances and trajectories for young people (e.g., Schoon and Lyons-Amos, 2016).

Young people’s growing involvement in status competitions in the pursuit of social status and success is aligned with extrinsic values embodied by a community of mutually interdependent MDC (Butler, 2018, 2021), which are evolutionary-based (Turke, 1990; Bernard et al., 2005; Cheng and Tracy, 2013; Maestripieri et al., 2017), developmentally salient (e.g., Elstad, 2010; Hamel et al., 2012), and culturally promoted (e.g., Kasser, 2002; Striegel-Moore and Bulik, 2007; Maestripieri et al., 2017; Luthar and Kumar, 2018). Crucially, this typology of status-seeking motives involves *display* and *promotion*; evolutionary researchers underscore that social status is dependent not only on the ability of the prestigious individual to confer benefits, but also on their advertisement of those abilities (Von Rueden et al., 2008). Social media is a potentially consummate socio-cultural medium for signaling MDC (Blake et al., 2018; Nesi and Prinstein, 2019; Butler, 2021), where young people and their broader ecology (e.g., parents; Luthar et al., 2020b) advance increasingly idealized or perfect exemplars of MDC to advance their social status and social success. As noted by Patricia Greenfield, young people’s prolific use of technologies in modern market cultures, exemplified by social media, develop individualistic values, behaviors and socio-cognitive skills that focus on aspirations such as enhancing one’s self-image to garner social recognition, status and fame (Greenfield, 2016).

In pursuing social status and success, young people are likely to learn from prestigious peers (i.e., horizontal cultural transmission) and non-related adults such as esteemed cultural figures from entertainment, fashion, and sporting industries (i.e., oblique cultural transmission; Mesoudi et al., 2016). Following these cultural transmission routes, young people’s identity-related processes become oriented toward *prestige bias learning* (Henrich and Gil-White, 2001; Jiménez and Mesoudi, 2019), and correspondingly, high-status ‘attractors’ (Barkow et al., 2012), who tend to embody idealized, flawless, or more extreme self-displays of MDC that are highly curated and often largely unattainable. In turn, young people deploy considerable information-processing capacities to negotiate social competition and apply culturally transmitted learning and knowledge to their efforts to achieve social status and success (Roth and Dicke, 2005; Balconi and Vanutelli, 2016). The most studied socio-cognitive processes in young people’s social media use, namely impression management, self-objectification, and social comparison, serve both evolutionary functions (Rosenberg, 1988; Rayo and Becker, 2007; Weimann et al., 2015) and are critical to optimizing one’s social position in individualistic and extrinsically oriented modern market cultures (Greenfield, 2016), as progenitors such as Goffman (1956) and Festinger (1954) fully realized. Young people’s application of their growing socio-cognitive and social competencies on social media are particularly sensitive to the dynamic of competitive self-enhancement among peers (Varella et al., 2014), and particularly responsive to fast-changing social conditions and culturally transmitted knowledge of modern market cultures, including cultural preferences, trends, and fashions (Baltes, 1997; Alexander, 2008; Sterelny et al., 2012).

Concomitant with the cultural promotion of status-seeking and associated extrinsic values, young people are obliged to develop an identity that is market-driven and embedded in self-narratives of success, status, and enhanced self-image (Butler, 2018, 2021). While grounded in a community of interdependent MDC, young people’s identity narratives developed over the course of adolescence and early adulthood will come to reflect their own personal experiences, to accommodate their own skills, limitations, and constraints, and to communicate their aspirations for the future (McAdams and Zapata-Gietl, 2015).

#### Young people’s pursuit of social status and success: self-optimizing socio-ecologies

The critical role that young people’s strivings for social status and success play in highly competitive and achievement-oriented forms of life is grounded in evolutionary, developmental, and socio-cultural influences. The work of evolutionary biologist and zoologist Richard Alexander and colleagues suggest that young people are developing in modern, market-based societies characterized by intensified within-species competition for resources (Alexander, 1974, 2008; Flinn and Alexander, 2007). The ecological dominance-social competition model proposes that, as humans became ecologically dominant, a within-species arms race for essential resources became the driving force in the evolution of large-scale modern societies (Flinn et al., 2005). Ecological dominance resulted in increasing social pressures—due to reduced mortality and population expansion— that selected for enhanced cognitive abilities, which, in turn, allowed hominins to become even more ecologically dominant, and so on. The need to influence other human beings and to succeed in social competitions arose not just between humans living in different groups, but also among humans living within the same group, because increased group-size meant intensified competition for resources within one’s own group (Alexander, 2008). In the wake of growing ecological dominance, traits that began to strongly covary with individual differences in survival and reproductive outcomes were those that allowed hominins to socially outmaneuver other hominins and achieve social success (Geary, 2005). As succinctly noted by one participant in a focus group study by Manago et al. (2008) on social media and identity, “Whenever you put any kind of information out there you have the intention of what you want people to think about you” (p. 450).

With the transition toward densely populated and urbanized market-based cultures over the past 200 years, young people’s evolved socio-cognitive abilities have been complemented by considerable parental and socio-cultural investments in their social productivity, social status, and success (Turke, 1990; Irons, 2005; Lancaster and Kaplan, 2010). From an evolutionary life-history perspective, parents have become implicated in a quantity-quality trade-off between number of offspring and the ability to invest in those offspring to optimize their chances for future success (Lawson and Mace, 2010; Giudice et al., 2015). Cultural shifts toward greater parental investment in fewer children allows parents to provide their children with opportunities to develop their *embodied capital* (e.g., physical health, intelligence and socio-cognitive abilities, social status, and social networks; Lancaster and Kaplan, 2010), and for children to profit from parental resources that will increase their chances to succeed (Kaplan, 1996; Worthman and Trang, 2018; Frankenhuis and Nettle, 2020). Increased population density and urbanization in modern market-based economies, contemporaneous with decreased mortality owing to improvements in public health (Lancaster and Kaplan, 2010), support the shift toward greater investment in fewer children indirectly through greater number of competitors. The increased number of competitors elevates the benefits of reproducing later and less often, because organisms and their offspring need to invest more in embodied capital to compete successfully (Frankenhuis and Nettle, 2020). Greatly reduced infant and child mortality rates markedly increases the probability that parental and broader social investments in children and young people will be realized in terms of productive and successful adulthoods (Lancaster and Kaplan, 2010).

When parents invest in their own or their children’s embodied capital to increase access to resources, they are simultaneously securing social rank and favorable positions in social networks, local communities, and society (Shenk et al., 2016). In the prestige-based forms of socio-economic and cultural competition characteristic of modern market cultures, young people’s motives to achieve social status and success in relation to their peers and broader communities is central (Alexander, 2008; Worthman and Trang, 2018; Winegard and Geary, 2021).

Consistent with this life-history view, in a study of the long-term impacts of fertility and socioeconomic position on descendant success in a Swedish cohort of 14,000 individuals born during 1915–1929, low fertility and high socioeconomic position predicted increased descendant socioeconomic success across four successive generations (Goodman et al., 2012). Modeling data on fertility declines worldwide, Shenk et al. (2016) conclude that the geographical areas marked by the greatest declines in fertility are those areas defined by modern labor markets with intense competition for jobs and an overwhelming diversity of consumer goods available to signal social status and well-being. These authors conclude that, as social competition becomes more focused on social climbing, couples invest more in material goods and achieving social status thereby influencing how many children they have.

The development of young people’s embodied capital extends beyond parental investment to include a broader socio-ecology that is designed to equip young people to become productive and successful adults (Kaplan et al., 2003; Lancaster and Kaplan, 2010; Worthman, 2012). During the initial stages of their development, young people’s embodied capital is nurtured by parents and extended kin, non-kin adults such as teachers, coaches, and counselors, and social institutions such as school and governmental provisions in areas like education and healthcare that vary within and across Western nations (Roser and Ortiz-Ospina, 2016; Schneider et al., 2021). An extended period of childhood with intensive parenting and alloparenting provides for the acquisition of socio-cognitive competencies and the evolution of coalitional networks necessary for young people to successfully negotiate the increasingly intense social competition of adolescence and adulthood (Alexander, 1990; Dunbar, 1998; Flinn et al., 2005; Flinn and Alexander, 2007; Kaplan et al., 2007). Progressing from adolescence through to young adulthood, young people transition from receiving parental and broader social investments in their embodied capital toward translating this embodied capital into their own lifetime productivity and life satisfaction, with benefits to society (Worthman, 2012). Young people’s prolific social media use during adolescence and young adulthood (Mir et al., 2019; Politte-Corn et al., 2023) straddles their progression from parental and societal investments in their embodied capital, to their own growing, strategic offline and online displays of status-enhancing embodied capital via MDC (Worthman, 2012).

Status competitions for limiting resources are hallmarks of modern market cultures (Alexander, 2008; Summers and Crespi, 2013; Winegard et al., 2018; Bernhardt et al., 2020), and the pursuit of status and admiration and respect of our peers have been deemed human universals (Anderson et al., 2015), constituting a fundamental evolutionary motive (e.g., Barkow, 1992; Bernard et al., 2005; Von Rueden et al., 2008; Schaller et al., 2017; Kenrick and Krems, 2018). Young people’s success in status competitions will bring tremendous social benefits: greater access to resources, greater respect, and greater ability to control social outcomes and satisfy preferred social goals as they mature (Geary, 2005; Von Rueden et al., 2008). Contested cultural resources that are part of young people’s developmental trajectory include their ability to attract mates (Ferguson et al., 2011), to garner status-enhancing material possessions (Jiang and Dunn, 2013; Parment, 2013; Bricker et al., 2014), to access prestigious educational placements and/or job training in the service of status attainment and future wealth (Deci and Ryan, 2012; Schoon and Lyons-Amos, 2016; Nelson and Dawson, 2017; Musselin, 2018), and the accrual of psychological resources such as self-esteem (Kavanagh and Scrutton, 2015), social recognition, and a sense of belonging (Baumeister and Robson, 2021). The importance of promoting one’s status to secure material and psychological resources is particularly relevant to individualistic and extrinsically oriented modern market cultures, where subjective well-being is more likely to be linked to achieving status (Kenrick and Krems, 2018) and to outperforming one’s peers in doing so (Latif, 2016; Präg et al., 2016).

Current developmental and neuroscientific research suggest that adolescence through to early adulthood is a pivotal period for status-seeking (Crone and Dahl, 2012). Young people’s status and identity-enhancing displays on social media disclose needs for positive social attention and belonging, peer recognition and approval, and ultimately, needs for social success (Gilbert, 2017). Their motivations for status attainment and peer admiration are mediated by culturally prominent extrinsic values, which have been intensifying over the last 40 to 50 years (Twenge J M, et al. 2010, 2012; Twenge J, et al., 2010; Twenge and Kasser, 2013). In their online signaling, young people are neurobiologically sensitive to both the extrinsic metrics that they signal to each other (Crone and Dahl, 2012), as well as to the profound social experiences of acceptance or rejection that emerge out of the medium’s capacities for feedback and reinforcement (Crone and Konijn, 2018; Meeus et al., 2019). Young people’s pubertal surge in testosterone levels which amplifies the motivational salience of social status in males and females, and that specifies the types of behavior and reward learning that result from this increased motivation, can vary widely across cultural contexts (Crone and Dahl, 2012). In highly competitive and achievement-oriented forms of life characteristic of Western or WEIRD societies (Henrich et al., 2010), young people’s use of social media is linked to their stage-salient motivations to attain status and secure their position in peer hierarchies (Somerville, 2013), and to their heightened sensitivities to and reliance on peer feedback and social rewards that center on extrinsic values, metrics and goal pursuits.

#### Self-optimizing socio-ecologies: young people’s status and identity-enhancement on social media

The medium of social media has quickly become part of the intense social competition of modern market cultures, where young people’s status and identity-enhancing signaling of MDC affords for, and amplifies, evolutionary based potentials for status-seeking (Gintis, 2011; Blake et al., 2018; Davis and Arnocky, 2020). Growing social inequality in many market societies is associated with increasing interpersonal competition accompanied by status anxiety, making status competitions over MDC more salient (Delhey and Dragolov, 2014; Buttrick et al., 2017). Social media provides young people with highly accessible, low-cost forums to signal their social status via optimal displays of MDC (Blake et al., 2018; Borgerhoff Mulder, 2018), helping them secure access to valuable social, psychological, and material resources that are limited, contested, and unequally distributed (Worthman and Trang, 2018). For example, examining the patterning of the entire population of “sexy selfies” (*N* = 68, 562) in females published from Twitter and Instagram during a one-month period in 2016 across 113 nations, the prevalence of sexy selfies was greatest in environments characterized by highly unequal incomes. Similar patterns across US cities and counties were found between indices of economic inequality and expenditures in women’s beauty salons and clothing stores.

Young people’s susceptibility to culturally promoted extrinsic values and goals influences their use of technologies such as social media, strongly encouraging them to engage in status and identity-enhancing signaling of MDC (e.g., Greenfield, 2016). In a pre-adolescent sample, fame was identified as the top cultural value, actively cultivated through social media use, providing young people with an audience to respond to and shape their desire for social recognition and status attainment (Uhls and Greenfield, 2012). In a Dutch survey study of undergraduate students, the pursuit of popularity and thus social status was the most potent predictor of Facebook use, exceeding needs for social stimulation, belonging, and a desire to learn about what friends are doing (Utz et al., 2012). The term social comparison and feedback seeking (SCFS) has been advanced to describe technology-specific behaviors that young people engage in on social media. SCFS enable young people to gain highly salient information related to MDC such as their appearance, behavior, and social status relative to their peers (Nesi and Prinstein, 2015; Nesi et al., 2017). Nesi and Prinstein (2019) advance the notion of *digital status-seeking* whereby young people engage in a set of online signaling behaviors (e.g., maximizing likes) to enhance their status and reputations. In their longitudinal study of young people’s use of platforms such as Facebook and Instagram, adolescents with greater reputations for digital status seeking reported more frequent social media use and greater adherence to extrinsic value criteria such as desires for popularity and status attainment.

Social media platforms often foster psychosocial environments where many young people feel pressured to adopt extrinsic ideals embodied by MDC, and to rely on others to validate their developing values, identities, and self-worth (Manago et al., 2008; Nesi and Prinstein, 2019). Cross-sectional and limited longitudinal and experimental data suggests that young people’s increased use of social media promotes greater investment in MDC such as materialism (Chu et al., 2016; Ozimek et al., 2017, 2024; Sharif and Khanekharab, 2017; Rai et al., 2018; Jameel et al., 2024) and physical appearance ideals (for review see Fardouly and Vartanian, 2016; Vandenbosch et al., 2022). Studies linking intensive social media use to increased materialism in young people includes samples of university students from evolving market economies in Saudi Arabia (Jameel et al., 2024), China (Chu et al., 2016) and Malaysia (Sharif and Khanekharab, 2017).

A scoping review investigating associations between social media use, body image and eating disorders among young people in 50 studies across 17 nations and multiple social media platforms, concluded that attention to social media as a risk factor for eating pathology clearly warranted attention outside of high-income WEIRD countries (Dane and Bhatia, 2023). Supporting the SEE framework, the evidence-base suggests that socio-cognitive processes linked to the competitive individualism of WEIRD economies such as thin-ideal internalization, self-objectification and social comparison, are also operative in more recently emerging Asian market economies (Dane and Bhatia, 2023). Prestige-bias learning appears to be evident as a form of cultural transmission in non-Western nations (Becker, 2004), and a recent systematic review found that young women in developing nations associate the thin appearance ideals portrayed in Western media with financial success (Thompson et al., 2020). At the same time, educated young women from China’s burgeoning market-economy were both influenced by globalizing Western body image norms, and interpreted these Western-based ideals in a manner sensitive to local norms and practices (Jackson et al., 2016).

While there is surprisingly little research on young people’s high achievement ideals and social media use, according to a Pew Research survey studying teens habits and experiences online, roughly 50% of young people surveyed endorsed posting about their accomplishments, the highest of all identified domains (Pew Research Center, 2018). Young people’s predilection to post about their accomplishments online is consistent with growing cultural pressures that they must succeed at the highest levels in all their pursuits, not just academically but also in athletics and other extra-curricular activities (Spencer et al., 2018; Curran and Hill, 2019; Ebbert et al., 2019; Luthar et al., 2020b). Remarkably, in the highly competitive and achievement-oriented forms of life that characterize modern market cultures, there has been very limited research into young people’s frequent postings of their achievements online.

Finally, according to evolutionary theory, social status in human societies is often based on prestige (Henrich and Gil-White, 2001; Cheng et al., 2010; Jiménez and Mesoudi, 2019), where prestigious individuals receive ‘freely conferred’ deference that is linked to others’ perceptions of their skills and characteristics. Young people allocate status in competitive online environments via *indirect reciprocity*, granting high status and esteem to peers who signal admirable displays or performances of culturally valued characteristics and competencies embodied by MDC (Summers and Crespi, 2013; Winegard et al., 2018). Signaling the most desirable displays of MDC yield special opportunities or benefits to the young person displaying them, such as establishing or enhancing their reputations as high achievers or winners, which in turn, may bring benefits to peers who associate with the prestigious young person. Young people’s online status and identity enhancing signaling of MDC has additional mutual benefits for signaler and receiver, since both participants learn how to hone their growing socio-cognitive skills and apply culturally transmitted knowledge to achieve social status and success. They will also learn how to cope with status rankings that may be difficult to change (Alexander, 2008), as they mature and progressively compete for limited and unequally distributed material, psychological and social resources.

As audiences observing online status competitions, young people learn how to pursue social status and success by looking at the profiles and behaviors of their peers, which provide critical situational cues and information about what is expected and appropriate when signaling online (Lease et al., 2002; boyd and Ellison, 2007; Antheunis and Schouten, 2011; Valkenburg and Peter, 2011; Ellison and boyd, 2013). For instance, young people often observe peers signaling a repertoire of enticing and highly desirable images and postings commensurate with status enhancement via optimal displays of MDC, which frequently lead to peer approval and positive feedback. Consistent with this notion, in a sample of young adult women (M = 19.6 years), more sexualized photos garnered more likes on Instagram than less sexualized photos, and women who posted more sexualized photos tended to get more likes in general and more friends and followers on both Instagram and Facebook (Ramsey and Horan, 2018). While tracking and evaluating each other’s online status and identity-enhancing displays, young people learn the nuances of how to signal and how to elicit and cultivate positive feedback and how to avoid negative feedback from their peers (Reinecke and Trepte, 2014; Singleton et al., 2016).

In their status and identity enhancing signaling of MDC on social media, young people pursue arenas of competition where they are most likely to be successful to optimize their social status and peer coalitional value (Summers and Crespi, 2013; Winegard et al., 2020; Butler, 2021). Consequently, young people will benefit substantially from knowing their own abilities and their best strategies for attaining and maintaining status and for responding to online peer evaluation and feedback. While social media constitutes an open-ended arena for status competitions in the pursuit of social status and success, soliciting and attracting valued peers, it also allows young people to display their worth as cooperators by forming peer coalitions even as they compete via their status and identity-enhancing performances. Young people may form coalitions and sub-coalitions based on complementary skills or interests, in many instances related to MDC or combinations of MDC. The formation of online status and identity-enhancing peer groupings accommodates enormous diversity in young people’s talents, characteristics, and preferences, and is flexible across time and space.

#### Young people’s “optimal” online status and identity-enhancing signaling moves toward the extremes

From an evolutionary perspective, young people’s status and identity-enhancing signaling of MDC is governed by *runaway social selection* (Flinn and Alexander, 2007; Crespi et al., 2022), involving arms race competitions among peers for social status. Social selection involves competitions with conspecific rivals which individuals must win to gain access to valued social, psychological, or material resources (Roughgarden, 2012). Decreasing constraints from natural selection, combined with increasing social competition, generates a potent runaway process in the evolution of social success (Flinn and Alexander, 2007). Cultural social selection can proceed under a runaway process, especially when young people choose to adopt and display culturally idealizing, perfectible, or extreme behaviors (e.g., Alexander, 1974; Flinn and Alexander, 2007; Boyd and Richerson, 2009; Legare, 2017; Lotem et al., 2017; Muthukrishna et al., 2018; Markov and Markov, 2020). Since young people’s status competitions involves establishing and maintaining relative superiority among each other (Alexander, 1974), the bar on extrinsic status indicators or MDC displayed on social media such as high achievements, physical attractiveness, or material success can be raised in a consistent direction generation after generation, in unending arms races (Crespi et al., 2022). In effect, competitive tendencies, and portrayals of MDC in the media and social media, can reciprocally influence one another similar to the process of gene–culture coevolution (Gintis, 2011).

Young people attempt to optimize their social status by signaling increasingly idealized (Pempek et al., 2009), perfectible (Curran and Hill, 2019), or more extreme embodiments of MDC on social media. For instance, concomitant with linear increases in perfectionism from 1989 to 2016 in United States, Canadian, and British samples of young adults, Curran and Hill (2019) underscore that incidence of body dysmorphia and eating disorders has risen by approximately 30% among late adolescent girls since the advent of social media (e.g., Smink et al., 2012; PwC, 2015), with increasing numbers of young females turning to plastic surgery as an image-enhancement tool (e.g., British Association of Aesthetic Plastic Surgeons, 2015; Thomas, 2015; American Society of Plastic Surgeons, 2016). Recent data suggests that higher levels of social media use elevate young people’s concerns about their physical attractiveness in both males and females (for narrative review including studies on Facebook, Instagram, TikTok and Snapchat, see Vandenbosch et al., 2022). These trends suggest that young people and particularly young females are responding to pervasive cultural pressures to optimize online displays of their physical appearance as a key means of elevating their status and self-worth among their peers (Blake et al., 2018; Vandenbosch et al., 2022).

The forms of competition that are characteristic of individualistic and extrinsically oriented modern market cultures have changed progressively from the dominance-based physical aggression of earlier societies to a more prestige-based form of competition based on socio-economic and cultural conditions and goals (Winegard and Geary, 2021). The attainment of prestige-based competencies and associated gains in social status and success shift selection pressures from favoring traits associated with physical dominance, to the cognitive abilities and academic motivations that contribute to achievement in school and the development of culturally valued characteristics and abilities (Winegard and Geary, 2021). For instance, the pervasive use of high achievement testing across modern market economies has been accompanied by widespread emphases on student and institutional rankings to identify both the most capable students, and the most prestigious academic environments (see Butler, 2021 for review). Consequently, young people, their parents, and school personnel focus their efforts and resources on maximizing young people’s high-stakes test performance, making crucial links between young people’s test scores and their prospects in the future for lucrative employment and material success (e.g., Alon and Tienda, 2007; Buchmann et al., 2010).

Studying the highly competitive and achievement-oriented socio-ecologies of high-achieving schools, young people’s social media use suggests high levels of social comparison related to status-anxieties linked to MDC such as high academic and extra-curricular achievement, physical attractiveness, and displaying signs of material success (e.g., clothes; Luthar et al., 2013; Luthar and Kumar, 2018; Ebbert et al., 2019; Luthar et al., 2020a). In one of the few studies that include both youth and parental social media use, while young people engage in high levels of social comparison on social media to help determine their competitive standing, parents use social media as a platform to signal their children’s prestigious achievements and to enhance their social standing within the community (Luthar et al., 2020a).

In complementary qualitative studies, young people’s experience of high achieving schools in Australia (Spencer et al., 2018) and highly competitive equestrian training programs in Sweden (Broms et al., 2022), samples of mostly adolescent females describe competitive pressures to display identities encapsulated by the terms “*super girl*” and “*super equestrian*.” The *super girl* pursues entrance into an elite college as her ticket to elevated status and future wealth, while the *super equestrian* signals the most desirable online identity, namely a rider who is attractive, wears the ‘right clothes’, is successful, and acts ‘professionally’. While young people’s displays of increasingly idealized, perfect, or extreme versions of culturally valued characteristics and competencies need not be adaptive, they do allow peers and broader audiences to judge which young people have achieved the most optimal result and therefore accord status as a social outcome (Summers and Crespi, 2013).

In summary, young people exist in a fast changing and increasingly complex, competitive, and achievement-oriented global world that informs their developing identities and pursuit of social success. Progressing from adolescence to young adulthood, young people transition from receiving parental and broader social investment in their embodied capital toward investing their embodied capital into their own economic productivity and life satisfaction. Across this developmental window, young people engage in status and identity-enhancing signaling on social media to cultivate their social status, and to help secure access to psychological, social, and material resources integral to their identity and well-being. Young people’s developing identities and goal pursuits have become interwoven into their use of social media, which in turn, are embedded in a cultural context that privileges extrinsic values and extrinsic markers of social success. Expanding on the SEE framework as a heuristic for understanding young people’s social media activity as it pertains to identity and self-making in modern market cultures, the next section delineates young people’s social media environments as extrinsically oriented social media econiches embodied by status and identity-enhancing market-driven criteria signaled in highly competitive and achievement-oriented forms of life.

### Part B: Young people’s participation in extrinsically oriented social media econiches in highly competitive and achievement-oriented forms of life

Research reviewed in Part A suggests that young people’s status and identity-enhancing signaling in competitive online environments will be motivated to advance their social status and achieve social success. Online platforms provide opportunities for young people to cultivate and display their embodied capital, and to develop socio-cognitive competencies that will become important psychological and social resources as they negotiate their places and make a living in the complex social worlds of adulthood. This section suggests that young people’s social media environments are constituted as dynamic ecological niches (Stendel et al., 2020), embodying and enacting their participation in competitive and achievement-oriented forms of life oriented toward the realization of extrinsic values and goal pursuits, as their online activities afford for status and identity-enhancement (Butler, 2018, 2021; Moreno and Uhls, 2019).

Social media can be viewed as an ecological niche where organism and environment effectively produce one another in a process of mutual constitution (Stendel et al., 2020). Young people’s extrinsically oriented social media niche is evolving over time, co-constructed by the interaction of inherited predispositions for status-seeking and culturally transmitted norms, values, practices and metrics that pertain to social status and social success. At once evolutionary-based and market-driven, this process of cultural evolution may (re)produce the ecological structures that afford for and amplify genetic predispositions for status-seeking in socially competitive environments (Maestripieri et al., 2017). The term “market-driven” encapsulates the social, cultural and technological influences that shape and amplify the expression of evolutionary-based status motives in modern, market cultures.

An ecological niche is an animal’s position in an ecosystem that affords for the resources it needs to survive (Ramstead et al., 2016). It is a distinctive way of life (Gibson, 1979; Odling-Smee et al., 2003; Sterelny et al., 2012; Fuentes, 2014) that provides for a set or landscape of affordances (Rietveld and Kiverstein, 2014; Wagman, 2019). Niche construction theory describes how organisms modify their niches over time and thereby shape the external conditions of their existence with evolutionary and ecological consequences (Lewontin, 1985; Odling-Smee et al., 2003; Sultan, 2015). The construction of an extrinsically oriented social media econiche scales up across young people over time, generating directional and potentially stable alterations in the social media environment toward affordances that are consonant with, and accentuated by, highly competitive and achievement-oriented forms of life (Laland and O’Brien, 2010). For example, longitudinal data show that designer changes and innovations in the functional affordances provided by social media platforms, which foster highly visual and highly enticing image-based social media sites such as Instagram or Snapchat accentuating image enhancement and extrinsic values, have become more prominent in young people’s lives (Rideout and Robb, 2018).

An affordance is “what it offers the animal, what it provides or furnishes, either for good or ill” (Gibson, 1979, p. 127). Affordances are “things” of our everyday environment which have perceivable psychological value for us, regarding what possibilities they offer our actions and more broadly our intentions and our goals (Heft, 2003). Perceiving and acting on affordances are therefore best understood as value-realizing activities (Hodges and Baron, 1992). In the value-rich ecology of social media, extrinsic values provide an emergent organization for the young person-environment relation constituted on social media platforms that facilitate status and identity-enhancing signaling. In their intersubjectively shared extrinsically oriented social media econiche, the perception of affordances by the young person and the perception of affordances by the peer group are underwritten by the same principles (Marsh et al., 2006; Wagman et al., 2018). Young people are sensitive to hierarchically nested affordances and to the hierarchically nested affordances made available to each other (Wagman et al., 2018). Young people engage with these hierarchical nested affordances as an emergent, higher-order complex particular- namely a singular but multifaceted and multileveled higher-order relationship (Wagman and Stoffregen, 2020).

In young people’s extrinsically oriented social media econiche, affordances for status and identity-enhancement are superordinate to affordances for intrinsic needs such as recognition, belongingness, and self-worth, which are subordinate to, and nested within extrinsic pursuits and social practices co-extensive with highly competitive and achievement-oriented forms of life. For example, young people’s status and identity-enhancing signaling of MDC encourages an ecology of impression management and social comparison among peers, where dissemination of more idealized and objectified self-displays has utility, and dissemination of complex or balanced self-displays that incorporate feelings of inadequacy, insecurity, and self-doubt, largely do not (Reinecke and Trepte, 2014). Congruent with online self-enhancing presentation norms, in a six-month longitudinal study of young adults “authentic” versus “inauthentic” self-presentations, “authentic” self-presentations were associated with benefits to well-being, but only when authentic self-presentation was congruent with shared online expectations that young people communicated positive experiences and positive affect to their peers (Reinecke and Trepte, 2014). Relatedly, young people often focus on extrinsically oriented content that makes them look good to each other (Lenhart, 2015; Niland et al., 2015), fashioning self-narratives as they wish to be regarded by their peers and communicating this enhanced self-image easily and often to a wide variety of others (Manago et al., 2008; Liu et al., 2016).

Notwithstanding the superordinate position of status and identity-enhancement, the relationship among online affordances is flexible and allows for the play of extrinsic and intrinsic registers. Any given MDC, such as signaling a trendy consumer possession, a particular happy peer encounter, or a physically attractive image of oneself, can have multiple affordances (e.g., status and identity enhancement, sense of belonging), and, in complementary fashion, any given affordance, like affording for status and identity-enhancement or belonging, can be actualized by different MDC (e.g., physical attractiveness, high achievements; material success; see Wagman, 2019). From the SEE perspective, meaning in young people’s social media environments may often emerge out of, or is enacted by, the socio-psychological play of extrinsic striving and intrinsic need.

At the same time, privileging extrinsic status and identity-enhancing affordances over intrinsic needs within a hierarchical, nested affordance structure creates challenges for young people. For example, from a neoliberal perspective, young people’s trends toward signaling increasingly idealized or perfect embodiments of MDC follows a market logic of growth and competition, where display of continuously improving culturally promoted extrinsic metrics are necessary to continue securing intrinsic needs for recognition, belongingness, and self-worth (Curran and Hill, 2019). From a mental health standpoint, substantial research shows that when individuals show disproportionate extrinsic relative to intrinsic values in their overall value structure, there is increased risk for mental health problems (e.g., Kasser, 2002; Dittmar et al., 2014; Luthar and Kumar, 2018; Van Den Broeck et al., 2019). See Figure 1B for young people’s self-optimization and positional self-making on social media.

#### Building personal and optimal models of social status and success online

A landscape of affordances refers to the total ensemble of available affordances for a population in any given environment or niche—it is the shared public environment of affordances (Bruineberg and Rietveld, 2014; Rietveld and Kiverstein, 2014; Ramstead et al., 2016). In their extrinsically oriented social media econiche, young people’s signaling of a community of mutually interdependent MDC make-up the predominant part of the affordance landscape, enacting highly competitive and achievement-oriented forms of life. While a landscape of affordances refers to all the affordances available to organisms in this form on life, the field of affordances emerges out of the landscape, made up the affordances to which an individual is particularly responsive to, that solicit their attention and action (Kiverstein et al., 2021).

On social media, young people are likely to be drawn to act on a specific field of affordances provided by MDC that are salient to them, building personalized and optimal models of social success as a function of their concerns, organismic states, traits, and abilities (Bruineberg and Rietveld, 2014; Rietveld and Kiverstein, 2014). For instance, some young people may be drawn to the status and identity-enhancing affordances offered by postings centered on physical attractiveness and high extracurricular achievements, while other young people may be oriented toward displays of popularity or status-enhancing possessions. Young people’s engagement with, and competition for (Reed, 1993; Cisek, 2007), status and identity-enhancing affordances offered on social media point two ways: to their attributes, skills, and resources coupled with the field of affordances that is oriented toward their self-optimization; and to the environment of socio-cultural values and practices that permeate the landscape of affordances, constituting their extrinsically oriented social media niche that they share and construct with other young people (Petrusz and Turvey, 2010; Kiverstein et al., 2021).

#### Young person-social media environment coupling: promoting social status and success

In young people’s social media econiches, the intensification of their status and identity enhancing signaling is often informed by accelerating, positive feedback processes that move toward progressively more idealized, perfect or relatively extreme metrics of social success. Young people’s exchanges on social media afford for countless signaling opportunities of prestige MDC in densely rewarding peer contexts (Blake et al., 2018; Butler, 2018, 2021). MDC constitute different arenas where young people compete in status competitions (Barkow, 1989; Barkow et al., 2012) and express their identities and aspirations for social success, by signaling culturally valued characteristics and competencies embodied by MDC within their social media environments.

The self-accelerating, positive feedback cycles of status and identity enhancing signaling are made possible by the speed, scale, range, and volume of social exchanges afforded by highly rewarding and enticing social media platforms (Nesi et al., 2018a), whose functional architecture is embedded in culturally prominent extrinsic values (DeVito et al., 2017; Rideout and Robb, 2018). Young people adapt technological affordances such as asynchronicity, visualness, and quantifiability (Nesi et al., 2018b) to curate and display self-focused embodiments of MDC directed toward status and identity enhancement (Treem and Leonardi, 2013; Ellison and Vitak, 2015; Nesi et al., 2018a; Moreno and Uhls, 2019; Butler, 2021). For example, they post status updates (e.g., Twitter and/or Facebook), videos (e.g., YouTube), and photos or “selfies” (e.g., Instagram and/or Snapchat) that highlight positive or interesting features of the self in order to gain attention or admiration from their peers (Sung et al., 2016; Meeus et al., 2019). The medium also affords for entirely novel online behaviors such as enhancing one’s status among peers through opportunities to accumulate “likes” and “followers.” For the first time, the quantifiability of social media provides numerical indicators to measure status that can be effortlessly counted, compared, and viewed by a wide range of expectant peers (Nesi et al., 2018a).

Developmentally salient and culturally promoted metrics often focus young people’s attention and expectations toward their own and each other’s reputations and status in relation to how high they achieve, how good they look, and how their materialistic displays reflect their social success and developing selves. Social media transforms the scope and scale of comparative processes, whereby young people engage in upward and judgmental social comparisons of their body image, high achievements, or status-enhancing possessions not only with their immediate community of peers, but with virtual celebrity models or social media influencers whose self-displays are the product of extensive image manipulation and enhancement (Ho et al., 2016; Charmaraman et al., 2021; McComb and Mills, 2022; Pedalino and Camerini, 2022). Young people are aware of the quantifiable metrics employed by their peers as feedback to their status and identity enhancing displays, and even the time it takes to accrue those markers (Madden et al., 2013; Chua and Chang, 2016). A qualitative study of Facebook use in young people 15–19 years of age found that young people’s communicative and social practices online were tied to the technological affordances of the platform, such as the prominence of visual images to represent identity and the use of metrics such as ‘likes’ and ‘friends’ to negotiate identities and relationships (Pangrazio, 2019). Additional design features that solicit young people’s attention and promote emotional engagement when sharing status and identity-enhancing information online include status updates, wall posts and group membership (Tong et al., 2008; Haferkamp et al., 2012; Lindström et al., 2021), as well as the ability to continually scroll and replay desired and identity-compatible content features (Noë et al., 2019). In the process of facilitating status and identity-enhancing signaling, technological affordances provided by social media platforms enact relations between status metrics and intrinsic needs (e.g., belongingness, self-worth) that may shape young people’s development and self-making in pervasive and powerful ways (e.g., Brinkman et al., 2020).

Critically, status and identity-enhancement are enacted by a sender-receiver relation in response to affordances provided by young people’s signaling of MDC. These affordances are nested within a social structure and set of socio-cultural practices that fosters extrinsic values, where a young person’s signaling of their preferred MDC may be reliably met with recognition and validation, often very rapidly and by many peers. This person-environment ecology currently puts in place a very powerful nexus of dense peer reinforcement for status-seeking and identity enhancement when young people signal in response to affordances provided by MDC. Status and identify enhancement and are not inherent in either the young person or the social media platform but found only in the young person-social media environment system (Chemero, 2013).

## Summary and conclusion

This paper has examined young people’s social media use from the SEE approach, which proposes that the interaction of evolutionary based motives for status-seeking, and the continuing development of individualistic and extrinsically oriented market-based societies, has led to the growth of highly competitive and achievement-oriented forms of life. The highly competitive and extrinsic value orientation of modern market cultures shapes and constrains young person-social media ecologies, operating on socio-cultural and interpersonal contexts (e.g., accentuating strivings for status), individual psycho-social processes (e.g., fostering impression management, self-objectification, and social comparison), and institutional interests (e.g., designer features that encourage short-term attention, metrification and dense reinforcement, combining to drive platform use). Social media is both continuous with and extends young person’s learning environments that are constituted in complex modern market cultures. The distinct extensions afforded by and enacted in mediated communicative online environments transform cultural, interpersonal, and self-processes thereby shaping young people’s learning and development in new ways.

Modern market economies set distinct parameters for social success (Turke, 1990; Kaplan et al., 2003). Young people, their parents, and the broader culture track young people’s performance on MDC, including relationships to resource abundance and meanings within the broader social ecology. In young people’s extrinsically oriented social media econiches, young people and their socio-ecology will be sensitive to the status and identity-enhancing affordances offered by MDC, and young people will manipulate their signals in their communications with each other to optimize personal and social outcomes. Young people self-optimize on social media by cultivating personal pathways geared toward the pursuit of social status and success, acting on a specific field of affordances signaled by MDC that are salient to their interests, characteristics, abilities, and organismic states. Building personal models of success online, young people’s status and identity-enhancing signaling is influenced by prestige bias learning from high-status peers and esteemed cultural figures from entertainment, fashion, and sporting industries. Young people’s identity-related processes become oriented toward high-status ‘attractors’ (Barkow et al., 2012) who frequently model idealized, flawless, or more extreme self-displays of extrinsically oriented metrics (i.e., physical attractiveness, high achievements, material success), that are highly curated and often largely unattainable. In turn, young people deploy considerable socio-cognitive processing skills to negotiate social competition and apply culturally transmitted information to their efforts to achieve social status and success (Roth and Dicke, 2005; Balconi and Vanutelli, 2016).

Notwithstanding this emphasis on status achievement and social success, young people’s online signaling of MDC is multiplex (Barker et al., 2019), where affordances for status and identity-enhancement are superordinate to affordances for intrinsic needs such as recognition, belongingness, and self-worth, which are subordinate to, and nested within extrinsic pursuits and social practices co-extensive with highly competitive and achievement-oriented forms of life (Wagman, 2019). Young people’s personal agency becomes a crucial factor in their ability to negotiate the demands and pressures of modern market cultures, fostering pathways that enable them to attain status and success while developing a sense of purpose and future-orientation that resonates with their intrinsic values and pursuits, potentials, and skills. The overwhelming presence of social media in young people’s lives may partly be accounted for by the dynamic hierarchical and nested affordance structure of many social media environments, which supports complex self-development and meaning making via the interplay of extrinsic goal pursuits and fulfillment of their intrinsic needs.

Significant limitations in young people’s social media research currently constrain applicability of the SEE model. Although the SEE framework details a conceptual model for modern market cultures, the preponderance of data is still from Western nations and over-represented by well-educated young white people that are not representative of those young persons who use social media globally. There is substantial variation across studies in the specific MDC under study (e.g., appearance ideals vs. high achievements), as well as how receivers respond to young people’s self-displays, both concurrently and across time. As well, the developmental window from adolescence through to young adulthood spans roughly 14 years and concepts such status-seeking would benefit from greater precision across this broad developmental period.

Notwithstanding these limitations, SEE offers researchers a useful conceptual framework to guide research and test specific hypotheses about young people’s social media use. To begin, the SEE framework suggests that, across adolescence and emerging adulthood, young people’s self-optimizing displays and evolving self-narratives of success grounded in their preferred MDC come to express their aspirations and identities, as they cultivate psychosocial pathways co-extensive with competitive and achievement-oriented forms of life. Testing this conceptualization of young people’s social media use will benefit from person-centered, longitudinal studies that specify what MDC young people (and subgroups) signal to build optimal models of social success, while mapping how they incorporate peer feedback and evaluation into their ongoing self-focused displays and online behavior. This online self-making process will be influenced by, and hence ideally studied in relation to physical (e.g., puberty), socio-cognitive (e.g., adaptive versus maladaptive uses of self-objectification and social comparison), and ecological changes (e.g., moving from school to employment niches) that mark adolescence and emerging adulthood.

Second, the SEE framework may help contribute to our understanding of associations between young people’s more intensive use of social media and risk for experiencing internalizing mental health problems such as anxiety, depression, and low self-worth (Sarmiento et al. 2018; Meier and Reinecke, 2021). As described above, growing empirical research links young people’s use of social media for status-seeking and self-enhancement with vulnerabilities toward experiencing internalizing symptoms (Nesi and Prinstein, 2015; Meeus et al., 2019; Svensson et al., 2022). Tendencies to engage in upward or judgmental social comparisons over MDC, to place disproportionate emphasis on extrinsic vs. intrinsic goal pursuits, and to become dependent on or unduly influenced by peer evaluation and feedback, especially when this feedback is negative and occurs in competitive networks, are mechanisms that may translate status and identity-enhancing signaling in extrinsically oriented social media econiches into internalizing difficulties. Relatedly, young people’s social goals to increase their popularity and social status have also been associated with cyberbullying in cross-sectional and longitudinal research conducted in diverse countries (e.g., Wright et al., 2022).

Finally, evolutionary models that suggest young people’s online status or prestige is linked to building status and identity-enhancing coalitions in a networked exchange system (Winegard et al., 2018, 2020), where deference to peers signaling culturally valued MDC benefit both the prestigious individual and the coalitional peers who become allied with them. Following from this, it would be important to more fully investigate the perceptions of young people who alternately assume roles as senders, receivers and audience members in social media networks, to determine whether young people see themselves as members of online peer coalitions, and to investigate whether their responses to peer signaling are made partly to enhance their own social status in these respective roles. In short, the SEE model calls for further investigation of social media as a network which promotes extensive status and identity-enhancing interactions as young people develop their selves in the highly competitive and achievement-oriented forms of life characteristic of modern market cultures.

## Data availability statement

The original contributions presented in the study are included in the article/supplementary material, further inquiries can be directed to the corresponding author.

## Author contributions

SB: Conceptualization, Writing – original draft, Writing – review & editing.
